# Supplementary material for: Enhanced specificity mutations perturb allosteric signaling in CRISPR-Cas9
Source: eLife. 2021 Dec 15;10:e73601. doi: 10.7554/eLife.73601 (PMC8741213; doi:10.7554/eLife.73601)
Supplement: Transparent reporting form [file elife-73601-transrepform1.docx]

***eLife’s* transparent reporting form**

We encourage authors to provide detailed information *within their submission* to facilitate the interpretation and replication of experiments. Authors can upload supporting documentation to indicate the use of appropriate reporting guidelines for health-related research (see [EQUATOR Network](http://www.equator-network.org/%20)), life science research (see the [BioSharing Information Resource](https://biosharing.org/)), or the [ARRIVE guidelines](http://www.plosbiology.org/article/info:doi/10.1371/journal.pbio.1000412) for reporting work involving animal research. Where applicable, authors should refer to any relevant reporting standards documents in this form.

If you have any questions, please consult our Journal Policies and/or contact us: [editorial@elifesciences.org](mailto:editorial@elifesciences.org).

**Sample-size estimation**

- You should state whether an appropriate sample size was computed when the study was being designed
- You should state the statistical method of sample size computation and any required assumptions
- If no explicit power analysis was used, you should describe how you decided what sample (replicate) size (number) to use

Please outline where this information can be found within the submission (e.g., sections or figure legends), or explain why this information doesn’t apply to your submission:

- Three molecular dynamics (MD) replicates of ~1.2 µs each were run for each of the simulated systems (i.e., the CRISPR-Cas9 complex as WT and including the K855A, K848A and K810A mutations, as well as the isolated HNH domain as WT and including the three K-to-A mutations, resulting in a total of eight systems).

- Analysis of the results has been carried out on the separated replicas (Figs. S2, S4-S8), and for the overall ensemble (main text and Fig. S7-S9).

- µs-length replicates displayed convergent dynamical motions for the CRISPR-Cas9 system (Figs. S2, S4-S9). This provided a solid ensemble reproduced well across replicas, which motivated our choice of three replicates of 1.2 µs each.

- Replicate numbers for NMR experiments were determined from experience, practical considerations, and consistency with the field. Replicate relaxation time points in dual-field NMR studies were kept consistent for all samples.

**Replicates**

- You should report how often each experiment was performed
- You should include a definition of biological versus technical replication
- The data obtained should be provided and sufficient information should be provided to indicate the number of independent biological and/or technical replicates
- If you encountered any outliers, you should describe how these were handled
- Criteria for exclusion/inclusion of data should be clearly stated
- High-throughput sequence data should be uploaded before submission, with a private link for reviewers provided (these are available from both GEO and ArrayExpress)

Please outline where this information can be found within the submission (e.g., sections or figure legends), or explain why this information doesn’t apply to your submission:

- Three MD replicates of ~1.2 µs each were run for each of the simulated systems

- Analysis of the results has been carried out on the separated replicas (Figs. S2, S4-S8), and for the overall ensemble (main text and Fig. S7-S9).

- NMR relaxation experiments were performed at two magnetic field, 600 and 850 MHz, as stated in the Methods and Fig. 4 caption. Error bars in NMR relaxation data were determined from replicate relaxation points, at least 3-4 per sample. The relaxation points themselves, along with replicates, are listed in the Methods section.

**Statistical reporting**

- Statistical analysis methods should be described and justified
- Raw data should be presented in figures whenever informative to do so (typically when N per group is less than 10)
- For each experiment, you should identify the statistical tests used, exact values of N, definitions of center, methods of multiple test correction, and dispersion and precision measures (e.g., mean, median, SD, SEM, confidence intervals; and, for the major substantive results, a measure of effect size (e.g., Pearson's r, Cohen's d)
- Report exact p-values wherever possible alongside the summary statistics and 95% confidence intervals. These should be reported for all key questions and not only when the p-value is less than 0.05.

Please outline where this information can be found within the submission (e.g., sections or figure legends), or explain why this information doesn’t apply to your submission:

- Raw NMR spectra are presented in Fig. 1. Analyzed chemical shift data, based on the raw spectra, are reported in Fig. 2, along with the equation for analysis and discussion of the 10% trimmed mean statistics (see Fig. 2 caption and Methods).

- Raw relaxation data are presented in Figs. 4 and S3, along with fits to exponential or hyperbolic tangent functions, as referenced in the Methods.

- Statistical analysis over MD replicates is reported in the Methods section and in the supplementary information.

(For large datasets, or papers with a very large number of statistical tests, you may upload a single table file with tests, Ns, etc., with reference to sections in the manuscript.)

**Group allocation**

- Indicate how samples were allocated into experimental groups (in the case of clinical studies, please specify allocation to treatment method); if randomization was used, please also state if restricted randomization was applied
- Indicate if masking was used during group allocation, data collection and/or data analysis

Please outline where this information can be found within the submission (e.g., sections or figure legends), or explain why this information doesn’t apply to your submission:

Not applicable

**Additional data files (“source data”)**

- We encourage you to upload relevant additional data files, such as numerical data that are represented as a graph in a figure, or as a summary table
- Where provided, these should be in the most useful format, and they can be uploaded as “Source data” files linked to a main figure or table
- Include model definition files including the full list of parameters used
- Include code used for data analysis (e.g., R, MatLab)
- Avoid stating that data files are “available upon request”

Please indicate the figures or tables for which source data files have been provided:

Analysis codes and script files can be downloaded from Github: <https://github.com/palermolab>. Resonance assignments for the HNH structure are available at bmrb.io under BMRB entry 27949.
